# Limiting the health impact of earthquakes: a call to action

**DOI:** 10.7189/jogh.16.03001

**Published:** 2026-01-12

**Authors:** Christian Popescu, Ryoma Kayano, Pierre Nabeth

**Affiliations:** 1 World Health Organization, European Centre for Preparedness for Humanitarian and Health Emergencies, Istanbul, Türkiye; 2 World Health Organization, Centre for Health Development, Geneva, Switzerland

## Abstract

Earthquakes are the deadliest natural hazards. While stronger building codes are essential for reducing their health impacts, achieving full compliance is a costly, long-term effort, particularly for low- and middle-income countries. We highlight low-cost, high-impact interventions across three areas that can complement building code implementation and significantly reduce risks. First, strengthening health emergency governance to ensure that lines of command and accountability are clear when a disaster strikes. Governments need response plans that outline actions, roles, and responsibilities, and enable coordinated action through emergency operations centres and incident command systems. Second, enhancing risk communication and community engagement is critical. Communities are often the first responders to seismic events. Training them in first aid and search and rescue, involving them in preparedness planning, and co-developing communication approaches ensure more tailored and actionable responses. Finally, improving international coordination can enhance the predictability and effectiveness of external support by establishing aid agreements, harmonising border-entry procedures, and ensuring staff are trained to work with and coordinate international Emergency Medical Teams. Governments and the international community should increase investments to limit the health impact of earthquakes, including through the outlined low-cost interventions.

## EARTHQUAKES POSE SIGNIFICANT RISKS TO POPULATION HEALTH

On 1 November 1755, a devastating earthquake struck Lisbon, Portugal. The disaster, occurring during the Age of Enlightenment, prompted a shift in public debate in Europe from viewing catastrophes as divine punishment to recognising the role of policy decisions, notably how Lisbon’s urban design intensified the destruction [1]. The idea that policy shapes disaster outcomes remains central to today’s approaches in Disaster Risk Reduction and is codified in the 2015 Sendai Framework for Disaster Risk Reduction [2]. It is in the same spirit that during the 77th World Health Assembly 2024, Member States of the World Health Organization committed to strengthening health emergency preparedness for disasters caused by natural hazards [3].

Earthquakes are the deadliest natural hazards, killing more than 700 000 people between 2000 and 2023 [4]. The immediate health impact of earthquakes, morbidity and mortality, is compounded by mid- and long-term consequences, including increased physical and mental health needs of survivors, displacements, disrupted access to health services, and environmental health challenges [5–8]. Another reminder of this occurred on 6 February 2023, when two earthquakes struck Southern Türkiye and Northern Syria, resulting in more than 50 000 deaths and over 100 000 injuries [9]. More than one year after the event, 40% of survivors still suffered from mental health conditions, while as many as 70% of those injured were expected to have long-term physical rehabilitation needs [10,11].

In this viewpoint, we argue that governments around the world can significantly limit the health impact of earthquakes, including through low-cost interventions feasible to implement in low- and middle-income countries (LMICs).

## NOT EVERY LESSON FROM PAST EARTHQUAKES IS EASILY IMPLEMENTED

Many lessons from past earthquakes have been learned and are well documented [12–14]. Yet, implementation remains challenging. The most important lesson is that enforcing strict building codes saves lives (‘earthquakes don't kill people, buildings do’) [15]. Retrofitting buildings to be earthquake-resistant is expensive. For Istanbul, Türkiye, for example, the estimated cost of strengthening approximately 90 000 high-risk buildings exceeds USD 19 billion [16]. For this reason, particularly in LMICs, implementing stricter building codes is a long-term process. Countries at risk should initiate this process by revising and enforcing building codes for new constructions while prioritising the retrofitting of hospitals and other critical infrastructure. Integrating safety features into new buildings, including health facilities, is highly cost-effective. In LMICs, it is estimated to increase overall investment needs by only around 3%, while saving approximately USD 4 for every USD 1 invested through reduced disruption and repair costs [17].

## LOW-COST INTERVENTIONS CAN ALSO SAVE LIVES

While structural safety remains a critical element, low-cost interventions can also significantly reduce risks. In this viewpoint, we want to highlight interventions across health emergency governance, risk communication and community engagement, and international coordination.

Health emergency governance is essential to ensure that lines of command and accountability are clear when a disaster strikes. Response plans facilitate this by outlining actions, roles, and responsibilities to expedite the response, including coordination through emergency operations centres and incident command systems, as well as the dispatch of medical surge support [18]. The World Health Organization provides guidance on developing such plans [19]. Experiences from earthquakes in Iran and Türkiye show that established plans greatly enhance the effectiveness of the health response [20,21]. Strengthening health emergency governance for earthquakes can be challenging, as earthquake response is inherently cross-sectoral and requires alignment across government authorities. Yet health crises, or even the threat of crises, can provide impetus for positive change. For example, Jordan leveraged the risk of cholera importation in 2022–23 to clarify the roles and responsibilities of various health actors within the sector and in coordination with other sectors [22].

A second priority is community engagement and risk communication. Communities are often the first responders and know their needs best. Therefore, they should be at the centre of efforts to limit the health impact of earthquakes [18]. In remote locations, communities might be the only assistance available immediately after an earthquake. During the 2015 earthquake in Nepal, for example, community health workers provided health services in remote areas days before external relief arrived [23]. Governments should engage communities in planning and enable them to provide first aid and search and rescue. A good practice is Sendai in Japan, near the epicentre of the 2011 Tōhoku earthquake, championing community-led approaches to make the city more disaster-resilient [24]. Challenges remain in risk awareness. In the European Union, for example, 35% of the population is exposed to seismic hazards while only 13% perceive themselves as at risk [25]. Governments should co-develop risk communication with communities and leverage trusted local influencers, such as faith leaders, to enhance awareness.

Lastly, the coordination of international support must be strengthened. Earthquakes can overwhelm even the strongest health systems, making international assistance indispensable. However, integrating external support into a national response introduces complexity. During the 2010 Haiti earthquake, for example, inexperienced volunteers and unsolicited donations led to duplication, coordination challenges, and delays in response [26]. Preparing to receive international support can mitigate this risk. Earthquake risk assessments can help estimate supply and service needs, such as trauma care materials and Emergency Medical Teams specialised in trauma, followed by supplies to sustain primary health services [27]. Governments should then engage with partner countries to develop aid agreements, making international support more predictable. Developing standard operating procedures for border authorities can expedite the entry of international support. By training officials to act as Emergency Medical Team Coordination Cells, countries can ensure that incoming teams are well coordinated [28].

Countries at risk should systematically monitor progress in reducing earthquake risk in the health sector. Established frameworks, such as those for monitoring the implementation of the International Health Regulations (2005), provide relevant indicators for planning, risk communication and community engagement, and for international support [29].

## A CALL TO ACTION

Although countries cannot prevent all loss of life from earthquakes, the associated health impact can be reduced, including through the low-cost interventions outlined in this viewpoint. In 2023, <1% of national budgets were allocated to risk prevention, and <1% of total official development assistance was allocated to disaster risk reduction, prevention, and preparedness [4,30]. Governments and the international community should increase investments in risk reduction to limit the health impact of earthquakes. Just as the 1755 Lisbon earthquake transformed thinking about disasters in Europe during the Age of Enlightenment, today’s evidence demands a similar shift.
